# Longitudinal brain structural alterations and systemic inflammation in obstructive sleep apnea before and after surgical treatment

**DOI:** 10.1186/s12967-016-0887-8

**Published:** 2016-05-17

**Authors:** Wei-Che Lin, Chih-Cheng Huang, Hsiu-Ling Chen, Kun-Hsien Chou, Pei-Chin Chen, Nai-Wen Tsai, Meng-Hsiang Chen, Michael Friedman, Hsin-Ching Lin, Cheng-Hsien Lu

**Affiliations:** Department of Diagnostic Radiology, Kaohsiung Chang Gung Memorial Hospital, Chang Gung University College of Medicine, Kaohsiung, Taiwan; Department of Neurology, Kaohsiung Chang Gung Memorial Hospital, Chang Gung University College of Medicine, Kaohsiung, Taiwan; Department of Biomedical Imaging and Radiological Sciences, National Yang-Ming University, Taipei, Taiwan; Department of Brain Research Center, National Yang-Ming University, Taipei, Taiwan; Division of Sleep Surgery, Department of Otolaryngology-Head and Neck Surgery, Rush University Medical Center, Chicago, IL USA; Department of Otolaryngology, Advanced Center for Specialty Care, Advocate Illinois Masonic Medical Center, Chicago, IL USA; Department of Otolaryngology, Kaohsiung Chang Gung Memorial Hospital, Chang Gung University College of Medicine, 123, Ta Pei Road, Niao Sung District, Kaohsiung, Taiwan; Sleep Center, Kaohsiung Chang Gung Memorial Hospital, Chang Gung University College of Medicine, Kaohsiung, Taiwan; Department of Biological Science, National Sun Yat-Sen University, Kaohsiung, Taiwan

**Keywords:** Obstructive sleep apnea, Voxel-based morphometry, Magnetic resonance imaging, Oxidative stress, Leukocyte apoptosis

## Abstract

**Background:**

Systemic inflammation, neurocognitive impairments, and morphologic brain changes are associated with obstructive sleep apnea (OSA). Understanding their longitudinal evolution and interactions after surgical treatment provides clues to the pathogenesis of cognitive impairment and its reversibility. In the present study, we investigate clinical disease severity, systemic inflammation, cognitive deficits, and corresponding gray matter volume (GMV) changes in OSA, and the modifications following surgery.

**Methods:**

Twenty-one patients with OSA (apnea-hypopnea index, AHI > 5) and 15 healthy volunteers (AHI < 5) underwent serial evaluation, including polysomnography, flow cytometry for leukocyte apoptosis categorization, cognitive function evaluation, and high-resolution brain scan. Disease severity, leukocyte apoptosis, cognitive function, and imaging data were collected to assess therapeutic efficacy 3 months after surgery.

**Results:**

Pre-operatively, patients presented with worse cognitive function, worse polysomnography scores, and higher early leukocyte apoptosis associated with increased insular GMV. There was reduced GMV in the anterior cingulate gyrus before and after surgery in the cases compared to that in controls, suggesting an irreversible structural deficit. Post-operatively, there were significant improvements in different cognitive domains, including attention, executive and visuospatial function, and depression, and in early leukocyte apoptosis. There was also a significant decrease in GMVs after treatment, suggesting recovery from vasogenic edema in the precuneus, insula, and cerebellum. Improvement in early leukocyte apoptosis post-surgery predicted better recovery of precuneus GMV.

**Conclusions:**

In OSA, increased disease severity and systemic inflammation can alter GMV in vulnerable regions. Surgical treatment may improve disease severity and systemic inflammation, with subsequent recovery in brain structures and functions.

**Electronic supplementary material:**

The online version of this article (doi:10.1186/s12967-016-0887-8) contains supplementary material, which is available to authorized users.

## Background

Obstructive sleep apnea (OSA) is characterized by repetitive episodes of apnea or hypopnea during sleep. Sleep fragmentation and intermittent hypoxia in OSA can trigger elevated oxidative stress and subsequent inflammation [1]. Several imaging studies have demonstrated structural and functional changes in the brains of patients with OSA, with corresponding neuropsychological impairments [2]. Treatments, such as continuous positive airway pressure (CPAP), can significantly improve memory, attention, and executive function that parallels correction of the structural deficits. These data can shed light for treatment approaches for OSA and serve as motivators for treatment adherence.

The role of peripheral blood inflammatory markers related to OSA has not yet been systematically investigated. Several systemic inflammatory markers are involved in endothelial damage that occurs during repetitive hypoxia/re-oxygenation injury [3]. The interface between the vascular endothelium and host inflammatory response may also be related to the activation of resident microglia and peripheral blood leukocytes [4], as well as the expression of inflammatory cytokines and adhesion molecules [5]. However, the role of these inflammatory markers in the pathogenesis of OSA, especially in its relationship with structural brain alterations and cognitive function, as well as the effects of different treatments on these markers, remains unclear.

Previous studies have demonstrated that direct oxidative damage associated with sleep deprivation occurs across many brain regions [6], and chronic intermittent hypoxia can increase brain cortical neuronal cell death [7]. Structural evaluation, such as voxel-based morphometry (VBM), is an assumption-free, objective, and operator-independent method that applies voxel-wise comparisons throughout the brain to detect differences in gray matter volume (GMV). Extensive morphological alterations in several brain areas in patient with OSA had previously been shown by VBM [8]. However, the reported structural changes in OSA have been highly variable due to different VBM pre-processing pipelines and statistical threshold settings used in studies. The therapeutic effect of CPAP on structural brain alterations is still not conclusive [9–11]. CPAP compliance varies from 28 to 80 % [12, 13] and there is a consensus that a certain number of OSA patients cannot or will not use CPAP. Surgery for OSA is not a substitute for CPAP, but a salvage procedure for those who have failed CPAP and other conservative therapies. No research to date has focused on the effects of surgery in OSA using the relatively optimized longitudinal VBM method.

Serial evaluation before and after surgical treatment, and comparison with data from healthy controls, may elucidate the underlying morphologic, inflammatory, cognitive, and clinical performance changes in OSA patients and is essential to improving therapeutic strategies. This study aimed to determine (i) if OSA patients demonstrate alteration of GMV after surgical treatment, (ii) if changes in GMV are affected by the baseline or changes of systemic inflammation and their relationship to disease severity after surgery; and (iii) if there is any correlation between GMV and cognitive performance before and after treatment.

## Methods

### Participants

This prospective study targeted patients with OSA [apnea-hypopnea index (AHI) ≥ 5] evaluated for surgical treatment. Twenty-one patients (18 men and 3 women; mean age, 40.14 ± 10.80 years; body mass index [BMI], 26.24 ± 3.40 kg/m^2^; AHI, 38.77 ± 19.91 events/hr) and 15 healthy volunteers (AHI < 5) (11 men and 4 women; mean age, 39.80 ± 9.53 years; BMI, 23.97 ± 2.50 kg/m^2^, AHI, 2.43 ± 1.61 events/hr) were recruited. The Hospital Ethics Committee approved the study and all of the participants provided written informed consent.

All of the control subjects and OSA patients, who had failed CPAP and other conservative therapies, were enrolled through the sleep center after presenting with snoring. All participants underwent similar evaluations, including clinical characteristics, overnight polysomnography (PSG), blood samples for assessment of leukocyte apoptosis, neuropsychological tests (NP), and magnetic resonance imaging (MRI) for brain structure calculation. The last three evaluations were done on the same day and within 2 weeks of PSG.

Overnight PSG was assessed according to a previous report [14]. All-night comprehensive diagnostic sleep studies were performed at the hospital’s sleep center. The severity of sleep-disordered breathing was classified according to the number of apneas and hypopneas during sleep, and was classified as normal for AHI 0–5, mild for AHI 5–15, moderate for AHI 15–30, and severe for AHI > 30 [15]. Central respiratory events were excluded from the severity classification. All PSGs were scored and read by a board-certified physician blinded to the study. Patients were excluded if they had a history of major mental disorder, brain injury or illness, diabetes mellitus, cerebrovascular disease, major cardiovascular disorder (e.g., stroke, heart failure, myocardial infarction), or central/peripheral disorders known to affect the autonomic nervous systems.

Concepts of surgical treatment for OSA are based on reducing the volume of redundant tissues, stiffening the flaccid soft palate, and suspending the collapsed tongue base to maintain airway patency in order to improve symptoms and reduce the sequelae of OSA. The efficacy and safety of multi-level surgery has been previously demonstrated [16]. All of the surgical procedures were performed by the co-corresponding author (H-C Lin) under general anesthesia, with orotracheal intubation. The techniques used were determined at the discretion of the treating sleep surgeon based on the severity of OSA by PSG, and upper airway abnormalities were examined by flexible fibroscopy. The surgical techniques were as previously described [17–19].

### Assessment of leukocyte apoptosis

Leukocyte apoptosis, and its subtypes, were identified according to a previous study [20] with APO 2.7-phycoerythrin (PE), early apoptosis, and late apoptosis. Positive APO 2.7-PE indicated apoptotic cells. Further analysis of early and late apoptosis was conducted by annexin V-FITC and 7-aminoactinomycin D (7-AAD). Late apoptotic cells demonstrated disruption of the cell membrane integrity while early apoptotic cells demonstrated early and reversible apoptotic changes. Results were expressed as percentages of specific fluorescence-positive cells.

### Neuropsychological tests

The NP battery of tests focused on attention, execution function, speech and language, and mnemonic and visuoconstruction abilities. Different domain evaluations were measured as in previous studies [21, 22] with subtests from the Cognitive Ability Screening Instrument (CASI) [23] and the Wechsler Adult Intelligence Scale (WAIS-III) [24]. The Beck Depression Inventory II (BDI), was used to evaluate severity of depression [25].

### MRI image acquisition

All cross-sectional and longitudinal volumetric structural MRI was performed on identical 3-Tesla GE Signa whole-body MRI systems (General Electric Healthcare, Milwaukee, WI, USA) equipped with an eight-channel head coil at the Kaohsiung Chang Gung Memorial Hospital in Taiwan. A T1-weighted three dimensional fluid-attenuated inversion-recovery fast spoiled gradient-recalled echo pulse sequence was used with following imaging parameters for each participant and time-point: TR/TE/TI = 9.5/3.9/450 ms; flip angle, 15°; NEX = 1; matrix size = 512 × 512; voxel size = 0.47 × 0.47 × 1.3 mm^3^; and 110 axial slices.

An experienced neuroradiologist, blinded to the participants’ status, visually examined all of the MR scans. None of the participants in the study were excluded.

### Imaging data analysis

*Cross*-*sectional VBM processing* To identify regional GMV differences between patients with OSA, before and after surgical treatment, and the healthy control group, structural T1-weighted images were processed using statistical parametric mapping (SPM8; http://www.fil.ion.ucl.ac.uk/spm; Wellcome Institute of Neurology, University College London, UK) and the VBM8 toolbox (http://dbm.neuro.uni-jena.de) with default settings as described in the manual. The procedure for the cross-sectional VBM pipeline followed that of previous cross-sectional based VBM studies from our group [26, 27] (additional details available in the Method of the online “Additional file 1”).

*Longitudinal VBM processing* The default longitudinal batch script in the VBM8 toolbox was used to identify longitudinal effects to GMV in patients with OSA before and after surgical treatment. In this pipeline the modulation step was not used because our focus was on relative tissue differences between different time-points within the same participant [27]. First, follow-up (after surgery) T1-weighted scan was registered to the baseline scan (before surgery). Second, the mean anatomic image was calculated using the realigned images and served as a reference image for realignment of baseline and follow-up scans for each participant. Third, the individual realigned baseline and follow-up scans were bias corrected to account for field inhomogeneities regarding the corresponding mean anatomical scan. Fourth, the resultant bias-corrected mean anatomical scan and realigned images were segmented into GM, WM, and CSF tissue segments using the VBM8 segmentation approach. Fifth, the DARTEL registration parameters were estimated using the GM tissue segments of the bias-corrected mean anatomical scan. The resulting registration parameters were applied to the corresponding tissue segments of the realigned baseline and follow-up anatomical scans. The resulting normalized GM segments for each time point for each participant were smoothed using an 8-mm FWHM Gaussian kernel and served as inputs for the subsequent longitudinal statistical model.

### Statistical analysis

#### Analysis of demographic data between groups

All statistical analyses of demographic data, including clinical profiles, laboratory data, global tissue volume, and NP tests, were performed using the independent *t* test, Pearson’s Chi square test, and analysis of covariance (ANCOVA), as appropriate (details of statistical analyses are noted in the legend of Table 1). The longitudinal data were compared in the patient group, before and after surgical treatment, using a paired t-test. All statistical significance was set at *p* < 0.05 (version 12, SPSS Inc., Chicago, IL, USA).

**Table 1:** Demographic characteristics of OSA patients and control subjects

	NC (controls)	OSA_baseline_ (before surgery)	OSA_follow_ (after surgery)	*p* value		
				NC vs. OSA_baseline_	NC vs. OSA_follow_	OSA_baseline_ vs. OSA_follow_
Number	15	21				
Sex (M:F)	11:4	18:3		0.418		
Age (years)	39.80 (9.53)	40.14 (10.80)		0.922		
BMI (kg/m^2^)	23.97 (2.50)	26.24 (3.40)	25.83 (3.73)	0.050	0.130	0.033*
HbA1c (%)	5.54 (0.25)	5.80 (0.46)	5.75 (0.54)	0.206	0.338	0.537
Sugar (mg/dl)	92.00 (8.04)	93.43 (12.14)	95.05 (12.40)	0.925	0.546	0.217
hs-CRP (mg/dl)	0.80 (0.81)	3.22 (4.30)	2.32 (2.62)	0.108	0.069	0.292
Total cholesterol	192.10 (34.93)	191.96 (29.98)	194.57 (32.00)	0.880	0.985	0.599
Triglyceride	120.87 (95.78)	156.13 (107.13)	154.87 (79.05)	0.441	0.343	0.926
HDL	62.67 (14.44)	54.70 (10.57)	53.39 (10.32)	0.085	0.056	0.393
LDL	106.40 (34.41)	122.04 (29.46)	115.04 (39.67)	0.171	0.540	0.318
Smoking (yes/no)	(14/1)	(18/3)	(19/2)	0.728	1.000	1.000
*Voxel-based morphometry*						
GMV (ml)	613.32 (49.33)	620.69 (72.14)	620.86 (72.69)	0.707	0.702	0.953
WMV (ml)	587.49 (59.16)	583.96 (53.91)	582.17 (50.92)	0.851	0.722	0.373
CSFV (ml)	216.52 (25.02)	215.96 (22.39)	216.81 (22.52)	0.943	0.971	0.477
TIV (ml)	1417.33 (113.22)	1420.61 (119.15)	1419.83 (119.06)	0.931	0.947	0.674
*Polysomnography (PSG)*						
AHI	2.43 (1.61)	38.77 (19.91)	25.21 (20.93)	0.000*	0.000*	0.004*
De-saturation index	0.77 (0.82)	26.59 (19.38)	16.88 (19.23)	0.000*	0.006*	0.009*
Average O_2_ saturation	97.09 (0.82)	95.59 (1.39)	95.81 (1.79)	0.004*	0.053	0.534
Snoring index	208.96 (240.67)	385.71 (179.17)	323.52 (181.58)	0.059	0.278	0.116
Systolic BP (mmHg)	124.90 (15.86)	135.58 (15.38)	132.07 (11.58)	0.538	0.497	0.323
Diastolic BP (mmHg)	75.53 (9.09)	84.58 (11.00)	82.55 (12.01)	0.082	0.142	0.469
Leukocyte apoptosis (%)						
Granulocyte APO 2.7 apoptosis	0.429 (0.219)	0.894 (1.012)	0.831 (0.940)	0.160	0.125	0.830
Monocyte APO 2.7 apoptosis	2.135 (1.410)	3.156 (3.321)	2.570 (2.903)	0.439	0.575	0.585
Lymphocyte APO 2.7 apoptosis	0.481 (0.226)	0.658 (0.444)	0.475 (0.255)	0.283	0.835	0.164
Total leukocyte APO 2.7 apoptosis	0.891 (0.426)	1.442 (1.084)	1.271 (1.004)	0.128	0.236	0.626
Granulocyte late apoptosis	11.726 (6.986)	16.667 (10.032)	14.599 (14.039)	0.107	0.331	0.622
Granulocyte early apoptosis	13.920 (5.615))	23.117 (11.007)	18.250 (12.008)	0.010*	0.302	0.099
Monocyte late apoptosis	12.593 (7.401)	12.480 (8.384)	14.520 (8.876)	0.936	0.428	0.318
Monocyte early apoptosis	15.263 (5.068)	23.254 (8.033)	16.796 (8.342)	0.002*	0.579	0.007*
Lymphocyte late apoptosis	2.331 (1.090)	2.306 (0.915)	2.577 (1.178)	0.906	0.647	0.601
Lymphocyte early apoptosis	5.410 (1.988)	6.716 (2.505)	6.600 (2.624)	0.147	0.210	0.981
Total leukocyte late apoptosis	6.875 (2.588)	7.541 (3.170)	7.972 (4.126)	0.407	0.174	0.744
Total leukocyte early apoptosis	9.203 (1.987)	13.535 (3.713)	10.599 (4.431)	0.000*	0.286	0.021*

Data are presented as mean (SD)

Age data was compared by independent t-test; sex data was compared by Pearson’s Chi square test; BMI data was compared by ANCOVA after controlling for age and sex; Sugar, HbA1c, hs-CRP, smoking, voxel-based morphometry measurement, polysomnography result and leukocyte apoptosis data were compared by ANCOVA after controlling for age, sex, and BMI

*AHI* apnea-hypopnea index; *ANCOVA* Analysis of Covariance;* BMI*, body mass index, *CSFV* cerebrospinal fluid volume; *GMV* gray matter volume; *HDL* high-density lipoprotein cholesterol; *hs-CRP* high sensitivity C-reactive protein; *LDL* low-density lipoprotein cholesterol; *NC* normal controls; *OSA* obstructive sleep apnea; *TIV* total intracranial volume; *WMV* white matter volume

NC vs. OSA_baseline_: Statistical comparison was performed by ANCOVA between controls and OSA_baseline_

NC vs. OSA_follow_: Statistical comparison was performed by ANCOVA between controls and OSA_follow_

OSA_baseline_ vs. OSA_follow_: Statistical comparison was performed by paired t test between OSA_baseline_ and OSA_follow_

* Statistical threshold was set at *p* < 0.05

#### Group comparisons of GMV

Cross-sectional (patient with OSA before/after surgical treatment vs. healthy control) and longitudinal (patient with OSA before and after surgical treatment) voxel-wise GMV differences were examined using ANCOVA (with age, sex, and BMI as covariates) and paired t-test with SPM8, respectively. To prevent possible partial volume effects around the border between different tissue segments, voxels with an absolute GM probability <0.2 were excluded in the statistical analysis.

Whole-brain statistical inferences were considered significant at family-wise error (FWE) corrected *p* < 0.05 using the cluster-extend threshold approach with 3dClusterSim in AFNI (Analysis of Functional NeuroImages, http://afni.nimh.nih.gov/afni/). Based on the results of the Monte Carlo simulation, a cluster size of at least 453 voxels and at least 639 voxels were considered statistically significant for the cross-sectional and longitudinal designs, respectively [3dClusterSim using the following parameters: single voxel *p* < 0.01, FWHM = 8 mm (cross-sectional)/9 mm (longitudinal) with GM mask and 10,000 simulations].

#### Correlation among GMV, clinical profile, leukocyte apoptosis, and NP tests

The mean GMV was extracted from the clusters, with significant statistical difference from the group comparisons, for further correlation analysis. Partial Pearson correlation analysis adjusted for age, gender, and BMI was performed to correlate GMVs showing significant differences in cross-sectional and longitudinal effect with clinical severity, leukocyte apoptosis, and NP tests. Significance was set at a Bonferroni corrected *p* < 0.05, accounting for multiple ROI comparisons.

## Results

### Demographic characteristics of the participants

Based on the demographic characteristics of the 21 OSA cases and 15 controls (Table 1), there were no significant differences in age, sex, or BMI between the OSA baseline (OSA_baseline_, before surgical treatment) and control groups. OSA_baseline_ had significantly higher AHI (*p* < 0.001), higher desaturation index (*p* < 0.001), and lower average O_2_ saturation (*p* = 0.004) during sleep, compared with controls. After surgery, the OSA follow-up group (OSA_follow_) still had significantly higher AHI (*p* < 0.001) and desaturation index (*p* = 0.006) compared with controls.

After surgical treatment, OSA_follow_ had significant improvements in BMI (*p* = 0.033), AHI (*p* = 0.004), and desaturation index (*p* = 0.009) compared with OSA_baseline_.

### Group comparisons of leukocyte apoptosis

Percentages of early apoptosis of total leukocytes (*p* < 0.001), granulocytes (*p* = 0.010), and monocytes (*p* = 0.002) were significantly higher in OSA_baseline_ than in the controls (Table 1).

In contrast, there were no significant differences in the percentages of any kind of leukocyte apoptosis between OSA_follow_ and controls.

Nonetheless, OSA_follow_ showed significantly decreased percentages of early apoptosis of total leukocytes (*p* = 0.021) and monocytes (*p* = 0.007) compared with OSA_baseline_.

### Group comparisons of NP tests

OSA_baseline_ had worse short term memory scores compared with controls (*p* = 0.048) (Table 2). OSA_follow_ also had improvements in BDI compared with controls (*p* = 0.048).

**Table 2:** Cognitive test results of OSA patients and control subjects

Neuropsychological tests	NC (n = 15)	OSA_baseline_ (n = 21)	OSA_follow_ (n = 21)	*p* value		
				NC vs. OSA_baseline_	NC vs. OSA_follow_	OSA_baseline_ vs. OSA_follow_
Attention						
Mental control (CASI)	9.53 (0.99)	9.40 (1.70)	9.50 (1.10)	0.664	0.908	0.776
Attention (CASI)	7.73 (0.59)	7.65 (0.81)	7.60 (0.68)	0.999	0.631	0.716
Orientation (CASI)	17.93 (0.26)	17.50 (1.82)	17.85 (0.49)	0.366	0.908	0.427
Processing speed (WAIS-III)	105.67 (18.36)	100.55 (9.24)	108.55 (11.81)	0.280	0.757	<0.001*
Executive function						
Digit symbol (WAIS-III)	10.73 (3.04)	10.15 (1.81)	11.50 (2.50)	0.458	0.563	0.001*
Abstraction (CASI)	9.73 (2.05)	10.25 (1.41)	10.70 (1.17)	0.645	0.145	0.119
Memory function						
Long-term memory (CASI)	10.00 (0.00)	10.00 (0.00)	10.00 (0.00)	1.000	1.000	1.000
Short-term memory (CASI)	11.01 (1.01)	10.06 (1.56)	10.67 (1.50)	0.048*	0.398	0.175
Speech and language function						
Language (CASI)	9.78 (0.46)	9.95 (0.22)	9.95 (0.22)	0.218	0.320	1.000
Verbal fluency (CASI)	9.27 (1.03)	8.95 (1.57)	9.30 (1.08)	0.265	0.550	0.330
Visuo-spatial function						
Picture completion (WAIS-III)	11.20 (2.04)	11.10 (2.15)	11.55 (2.70)	0.495	0.911	0.206
Letter number search (WAIS-III)	9.53 (3.27)	9.45 (2.09)	9.60 (3.27)	0.791	0.959	0.780
Block design (WAIS-III)	11.47 (3.36)	11.10 (2.43)	11.95 (2.91)	0.455	0.846	0.015*
Drawing (CASI)	9.87 (0.52)	9.95 (0.22)	9.90 (0.31)	0.693	0.901	0.577
CASI total score	94.85 (3.70)	94.01 (4.78)	95.47 (3.39)	0.357	0.809	0.163
BDI	8.47 (7.58)	8.50 (8.16)	4.70 (6.11)	0.675	0.048*	0.011*

Data are presented as mean (SD)

All data were compared by ANCOVA after controlling for age, sex, and BMI

*AHI* apnea-hypopnea index; *ANCOVA* Analysis of Covariance; *BDI* Beck Depression Inventory;* BMI* body mass index, *CASI* Cognitive Ability Screening Instrument; *NC* normal controls; *OSA *obstructive sleep apnea; *WAIS* Wechsler Adult Intelligence Scale

NC vs. OSA_baseline_: Statistical comparison was performed by ANCOVA between controls and OSA_baseline_

NC vs. OSA_follow_: Statistical comparison was performed by ANCOVA between controls and OSA_follow_

OSA_baseline_ vs. OSA_follow_: Statistical comparison was performed by paired t test between OSA_baseline_ and OSA_follow_

* Statistical threshold was set at *p* < 0.05

OSA_follow_ showed significant improvements in attention (processing speed, *p* < 0.001), executive function (digit symbol, *p* = 0.001), visuospatial function (block design, *p* = 0.015), and depression (BDI, *p* = 0.011).

### Group comparisons of gray matter volume (GMV)

**Fig. 1:**
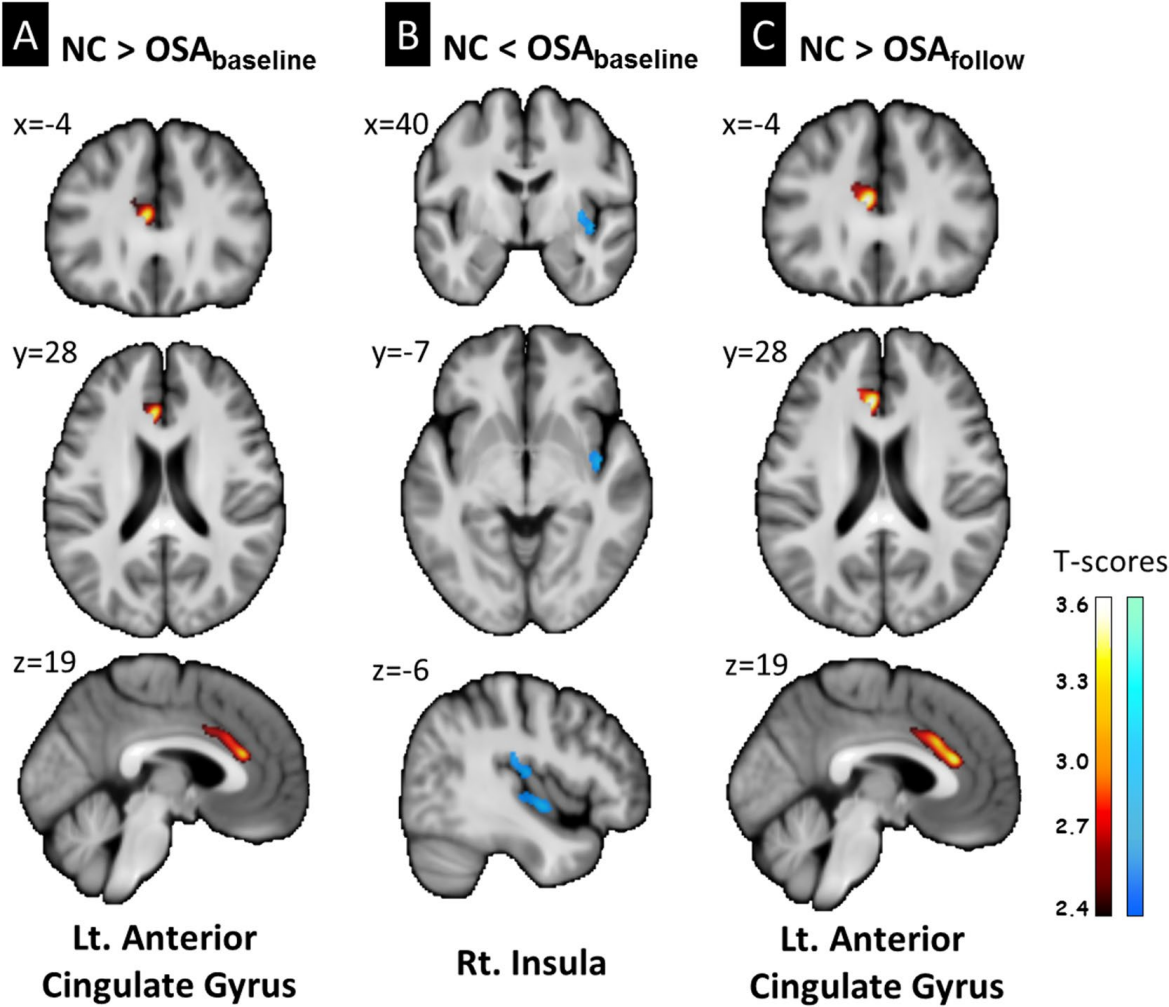
Regional *gray* matter volume differences between OSA_baseline_, OSA_follow_, and healthy control groups (corrected *p* < 0.05). Lt, *left*; Rt, *right*. For visualization, statistical parametric maps were superimposed on the participants’ mean T1 anatomical template for localization of significant gray matter volume changes

Regions of GMV difference between pre- and post-operative scans of patients and controls, and the longitudinal changes before and after surgery in the OSA group are presented in Table 3 and Fig. 1.

**Table 3:** Regions of GMV difference between pre- and post-operative scans of the patients and controls, and pre- and post-operative longitudinal changes in the OSA group

MNI atlas coordinates			Cluster size	Anatomical region	Brodmann area	t-score
x	Y	z				
Regional GMV decrease in OSA_baseline_ vs. NC						
−4	28	19	456	Left Anterior Cingulate Gyrus	24	3.41
Regional GMV increase in OSA_baseline_ vs. NC						
40	−7	−6	612	Right Insula	13	2.99
Regional GMV decrease in OSA_follow_ vs. NC						
−4	28	19	663	Left Anterior Cingulate Gyrus	24	3.65
Regional GMV decrease in OSA_follow_ vs. OSA_baseline_						
−24	−37	4	1047	Left Hippocampus (Hipp)	30	5.23
−7	−52	28		Left Posterior Cingulate Gyrus (PCG)	31	3.63
−24	−51	17		Left Precuneus	30	3.55
−49	4	−1	1566	Left Superior Temporal Gyrus (STG)	22	4.48
−42	29	−2		Left Inferior Frontal Gyrus (IFG)	47	3.86
−27	25	−3		Left Insula	13	3.61
Regional GMV increase in OSA_follow_ vs. OSA_baseline_						
−15	−81	−27	1254	Left Cerebellum	–	4.18

Gray matter volume (GMV) differences between groups are described in terms of MNI coordinates, voxel extent, brain side, and corresponding anatomical regions. The t-score of the voxel with the strongest group effect in a given cluster is also listed. The statistical criteria of the VBM results were set as a cluster level FWE corrected *p* < 0.05 (using a Monte Carlo simulation to correct the multiple comparison problem)

*FWE* family wised error; *Lt* left side; *MNI* Montreal Neurological Institute; *Rt* right side; *VBM* voxel-based morphometry

OSA_baseline_ showed significantly lower GMV in the left anterior cingulate gyrus and higher GMV in the right insula compared with controls. OSA_follow_ showed significantly lower GMV in the left anterior cingulate gyrus but not significantly higher GMV in the right insula when compared with controls.

**Fig. 2:**
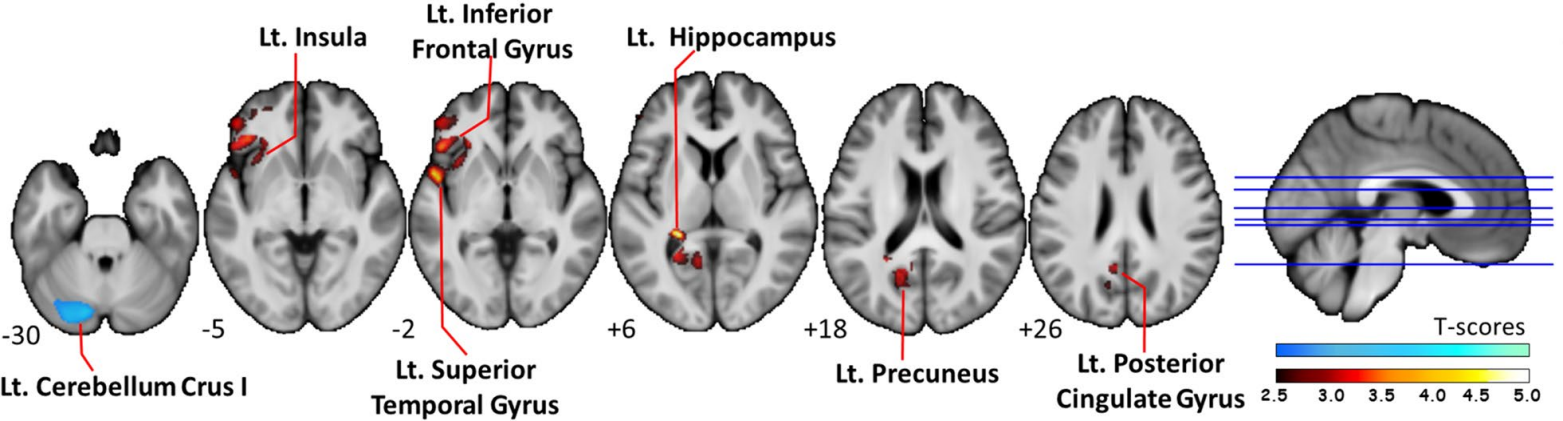
Regional *gray* matter volume differences between OSA_baseline_ and OSA_follow_ groups. Hot color map, OSA_baseline_ > OSA_follow_; cold color map, OSA_follow_ > OSA_baseline_ (Corrected *p* < 0.05)

An exploratory group-wise comparison of the OSA group before and after surgical treatment revealed that after surgery, patients exhibited decreased GMV in the left hippocampus/posterior cingulate gyrus/precuneus (Hipp/PCG/Precuneus) and left superior temporal gyrus/inferior frontal gyrus/insula (STG/IFG/Insula), and increased GMV in the left cerebellum (Fig. 2).

### Correlations between extracted regional GMV and all assessments

#### Correlations with clinical profiles

Before surgical treatment, worse PSG parameters (including higher AHI and desaturation index, and lower average O_2_ saturation) were associated with higher GMV in the right insula (*p*/*r* = 0.003/0.516; 0.003/0.508; 0.001/−0.574). After surgical treatment, there was no statistically significant correlation between PSG parameters and regional GMV (Table 4).

**Table 4:** Correlation between regional GMV and clinical disease severity (PSG parameters and leukocyte apoptosis)

Regional GMV differences between OSA_baseline_ and controls							
		AHI	Average O_2_ saturation	De-saturation index	Granulocyte early apoptosis	Monocyte early apoptosis	Total leukocyte early apoptosis
Left Anterior Cingulate Gyrus	r	−0.337	0.140	−0.217	−0.181	−0.259	−0.282
	p	0.059	0.444	0.234	0.321	0.153	0.117
Right Insula	r	0.516	−0.574	0.508	0.419	0.364	0.405
	p	0.003*	0.001*	0.003*	0.017*	0.041*	0.022*

Regional GMV differences between OSA_baseline_ and OSA_follow_							
		∆AHI	∆Average O_2_ saturation	∆De-saturation index	∆Granulocyte early apoptosis	∆Monocyte early apoptosis	∆Total leukocyte early apoptosis
Left Hipp/PCG/Precuneus	r	−0.035	−0.141	0.116	0.521	0.407	0.562
	p	0.885	0.565	0.637	0.022*	0.084	0.012*
Left STG/IFG/Insula	r	0.231	0.106	0.096	0.058	−0.199	−0.075
	p	0.341	0.665	0.696	0.812	0.415	0.760
Left cerebellum	r	0.201	0.311	0.167	−0.376	−0.438	−0.442
	p	0.410	0.195	0.495	0.113	0.060	0.058

Anatomical regions: Hipp/PCG/Precuneus, hippocampus/posterior cingulate gyrus/precuneus; STG/IFG/Insula, superior temporal gyrus/inferior frontal gyrus/insula

*AHI* apnea-hypopnea index; *GMV* gray matter volume; *OSA* obstructive sleep apnea; *PSG* polysomnography

* Statistical threshold was set at *p* < 0.05

Improvements in PSG parameters (follow-baseline) revealed no statistically significant correlation with regional GMV changes (follow-baseline).

#### Correlations with leukocyte apoptosis

Before surgical treatment, higher percentages of early apoptosis in granulocytes, monocytes, and total leukocytes were associated with higher GMV in the right insula (*p*/*r* = 0.017/0.419; 0.041/0.364; 0.022/0.405) (Table 4).

**Fig. 3:**
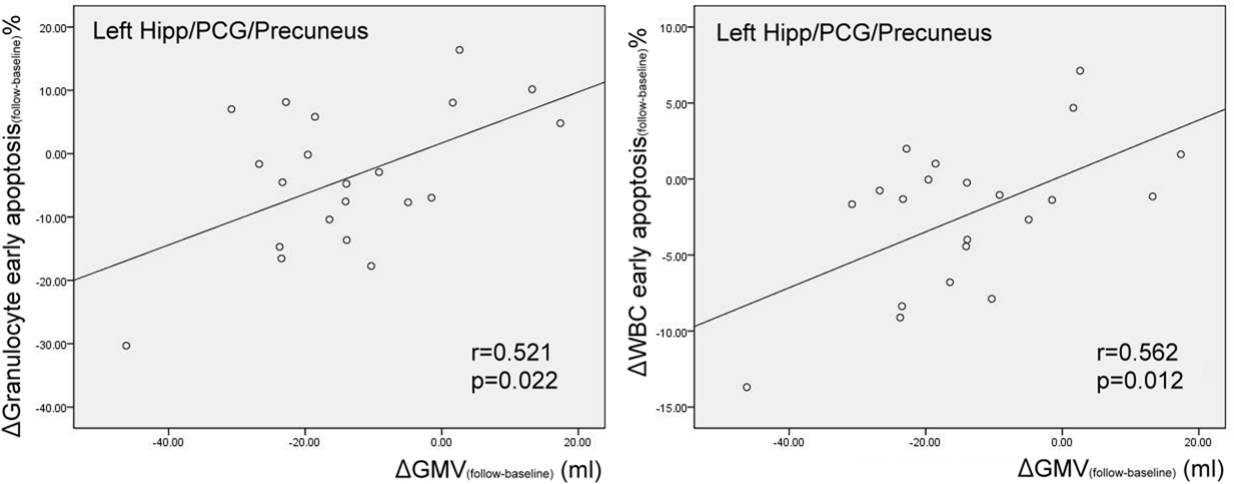
Correlations between interval change of leukocyte profile and regional *gray* matter volume

Improvements (follow-baseline) in early apoptosis of granulocytes and total leukocytes were significantly associated with recovery of GMV (follow-baseline) in the left Hipp/PCG/Precuneus (*p*/*r* = 0.022/0.521; 0.012/0.562) (Table 4; Fig. 3). Correlation between improvements (follow-baseline) in the early apoptosis of monocytes and total leukocytes, and recovery of GMV (follow-baseline) in the cerebellum tended to significance (*p*/*r* = 0.060/−0.438; 0.058/−0.442).

#### Correlations with NP tests

There was no statistically significant correlation between differences in regional GMV and NP tests.

After surgical treatment, OSA patients demonstrated improvements in cognitive function. However, there was no statistically significant correlation between changes in regional GMV (follow-baseline) and NP tests (follow-baseline).

## Discussion

### Summary

1. Longitudinal evaluation showed that surgery significantly improved AHI score, desaturation index, cognitive function, and decreased early leukocyte apoptosis.
2. OSA patients may undergo different types of GMV changes before and after surgery.
3. Before treatment, increased GMV in the insula was associated with increased disease severity and higher early leukocyte apoptosis. After treatment, recovery from increased GMV in pre-treatment, particularly in the Hipp/PCG/Precuneus, highly correlated with improved leukocyte early apoptosis.
4. OSA patients demonstrated persistently lower GMV in the anterior cingulate gyrus, even after surgical treatment.

### Peripheral leukocyte apoptosis before and after treatment

Changes in peripheral leukocyte apoptosis in OSA before and after surgery may partially explain the consequences and possible pathophysiology when patients experience repetitive episodes of hypoxia/re-oxygenation during the transient cessation of breathing [28]. Altered endothelial function is associated with enhanced release of specific leukocytes and systemic oxidative stress in OSA [29]. Previously, the use of CPAP has also been demonstrated to improve serum levels of C-reactive protein, tumor necrosis factor alpha, and interleukin-6, which can be further associated with extensive morbidity [30]. Increased early leukocyte apoptosis may be because some leukocyte injuries have not yet reached necrotic change [31] and provide the possibility of reducing activation of cell death receptors and mitochondria-dependent apoptotic pathways after aggressive treatment [32]. Like CPAP, surgical treatment can also alleviate the increased systemic inflammation by decreasing early leukocyte apoptosis in patients with OSA.

### GMV alterations before and after surgery

Although multiple pathophysiologies support the structural alterations occurring in OSA, differences in the studied patient population (sample size and disease severity), other co-morbidities, and different analysis procedures used with variable statistical criteria selection may lead to the differences observed between studies [8]. Structural changes in brain regions, including the frontal and parietal cortices, hippocampus, temporal gyrus, insula, cingulate gyri, caudate nucleus, and deep cerebellar nuclei, have been reported [9, 33, 34], and associated with different cognitive deficits [35]. According to previous reports [8], and this study, GMV reduction in the anterior cingulate gyrus before and after surgical treatment should be a hallmark of a patient with OSA. Physiologically, the cingulate gyrus, together with the insula, is mainly involved in cardiovascular control and may be damaged by intermittent hypoxia and sleep fragmentation.

Three months following surgery, a significant improvement in early leukocyte apoptosis was strengthened by the significant correlation with GMV reduction in the precuneus. However, the results here conflict with those reported in other related VBM studies in patients with OSA. O’Donoghue et al. showed no significant structural difference in patients treated with CPAP between pre-treatment severe OSA (mean AHI, 71.7 ± 7.0) and control subjects, and no changes following therapy [36]. Huynh et al. also reported no differences in GMV between patients with moderate-to-severe OSA and healthy controls, and no difference between CPAP and sham CPAP treatment [10]. However, Canessa et al. [9] reported a positive effect of CPAP treatment for severe OSA (mean AHI, 55.8 ± 19.1). After treatment, improvements in memory, attention, and executive-functioning paralleled GMV increases in hippocampal and frontal structures.

The present study enrolled OSA patients ranging from mild to severe disease severity (AHI, 38.77 ± 19.91; range, 13–75) and found longitudinal changes in GMV after surgery. This is partially consistent with Canessa’s observations. The difference in results may be explained by the different characteristics of the subject groups, different outcome measures and statistical criteria of imaging analyses (tissue volume vs. tissue concentration, uncorrected vs. corrected *p*-value), and different treatment effects between surgery and CPAP.

### Possible pathophysiology of GMV alterations

The mechanism of increased GMV before treatment and reduction after treatment can be explained by short-term or reversible cerebral vasogenic edema, a phenomenon also found in other hypoxic conditions such as high altitude cerebral edema and acute mountain sickness [37, 38]. An increased permeability of the blood brain barrier (BBB) tight junctions with subsequent formation of mild extracellular brain edema has been shown as the “normal” sequence of events under hypoxia [39]. Pre-treatment enlarged insular GMV positively correlating with increased hypoxic severity partially supports this hypothesis.

In the present study, elevated systemic leukocyte apoptosis before treatment in OSA may result from BBB breakdown, elevated peripheral mediators, and inflammation [40, 41]. In contrast, anti-inflammatory effects from interleukin, heat shock proteins, and adrenomedullin can improve BBB function [42]. We found that decreased granulocyte and total leukocyte early apoptosis were associated with GMV reduction in the Hipp/PCG/Precuneus. Furthermore, correction of increased GMV by reducing hypoxic exposure with surgery may re-normalize cerebral autoregulation and reduce subsequent leukocyte reaction. The results here support the possibility that cytokine-mediated BBB damage in cardiovascular complications of OSA may partially contribute to alterations in GMV.

The present study has its limitations. Although there is improvement of cognitive functions after surgery, the report fails to demonstrate the relationship between them and systemic inflammation and GMV alteration, presumably because of the small sample size of OSA subjects. Furthermore, statistically significant associations, especially in small groups, do not necessarily mean a causal relationship. Partially corrected hypoxic status (AHI score, Pre-OP 38.77 vs. Post-OP 25.21) and non-recovered brain injury (persistently decrease GMV in the anterior cingulate gyrus) after surgery may also explain the lesser improvement in neuropsychiatric functions. It is still too early to conclude on the long-term effects on brain structure, cognitive functions, and systemic inflammation from different treatments in OSA. Another limitation is that the healthy control subjects were not re-evaluated at 3 months, although changes in the GM of healthy participants over a short duration should be limited.

## Conclusions

OSA can alter GM integrity in vulnerable regions, an effect associated with increased disease severity and systemic inflammation. The possible interactions between systemic inflammation and GM changes may represent variant hypoxic patterns and their consequent processes. Surgical treatment can partially reverse structural brain abnormalities with reduced systemic inflammation. This study may serve as a motivator for better treatment adherence by patients.

## Additional file

10.1186/s12967-016-0887-8 The procedure for the cross-sectional voxel-based morphometry pipeline.

## Abbreviations

OSA: obstructive sleep apnea
GMV: gray matter volume
AHI: apnea-hypopnea index
CPAP: continuous positive airway pressure
VBM: voxel-based morphometry
GMV: gray matter volume
BMI: body mass index
MRI: magnetic resonance imaging
CASI: cognitive ability screening instrument
WAIS-III: Wechsler Adult Intelligence Scale
BDI: Beck Depression Inventory II
ANCOVA: analysis of covariance
