# Supplementary material for: Longitudinal brain structural alterations and systemic inflammation in obstructive sleep apnea before and after surgical treatment
Source: J Transl Med. 2016 May 17;14:139. doi: 10.1186/s12967-016-0887-8 (PMC4901987; doi:10.1186/s12967-016-0887-8)
Supplement: Supplementary file 1 — 10.1186/s12967-016-0887-8 The procedure for the cross-sectional voxel-based morphometry pipeline. [file 12967_2016_887_MOESM1_ESM.doc]

**The procedure for the cross-sectional voxel-based morphometry pipeline**

**Cross-sectional voxel-based morphometry processing**

The details of the cross-sectional voxel-based morphometry (VBM) pipeline was list as follows: First, to reduce the potential initial registration error, all native space volumetric structural T1-weighted images were reoriented with the origin set close to the anterior commissure. Second, all native space reoriented T1-weighted images were bias corrected and segmented into gray matter (GM), white matter (WM), and cerebrospinal fluid (CSF) compartments using the tissue-prior free segmentation approach in the VBM8 toolbox. This approach extended the unified segmentation model (1) by accounting for partial volume effects (2) based on the adaptive maximum aposterior approach (3), the spatially adaptive non-local means denoising filter (4), and the hidden Markov random field model (5). The overall global tissue volume for each tissue segment and total intracranial volume (TIV) were calculated using the native space tissue segments. Third, the native space GM and WM tissue segments were affine registered (12-parameter affine registration) to the Montreal Neurological Institute (MNI) template to remove the global brain size difference across the study participants. Subsequently, to achieve higher accuracy of inter-subject registration, the iterative diffeomorphic anatomical registration through exponentiated lie algebra (6) registration algorithm was used for the affined aligned GM and WM tissue segments. Non-linear deformations were used to modulate the participant’s GM tissue segments and interpolate to an isotropic voxel size with 1.5 mm. This modulation procedure allowed for inferences on relative GM tissue volume (adjusting for global brain size) rather than GM concentration for subsequent statistical analysis. After the above imaging processing procedure, covariate measurement provided by the VBM8 toolbox was used to check whether any artifacts, mis-segmentation problem, and registration error existed in the processed GM tissue segments. Lastly, the MNI space modulated GM tissue segments were smoothened using an 8 mm full width at half-maximum (FWHM) Gaussian kernel and served as input for the subsequent cross-sectional statistical model.

**Reference**:

1. Ashburner J, Friston KJ. Unified segmentation. NeuroImage 2005; 26: 839-851.

2. Tohka J, Zijdenbos A, Evans A. Fast and robust parameter estimation for statistical partial volume models in brain MRI. NeuroImage 2004; 23: 84-97.

3. Rajapakse JC, Giedd JN, Rapoport JL. Statistical approach to segmentation of single-channel cerebral MR images. IEEE Transactions on Medical Imaging. 1997; 16(2):176–186.

4. Manjon JV, Coupe P, Marti-Bonmati L, Collins DL, Robles M. Adaptive non-local means denoising of MR images with spatially varying noise levels. J Magn Reson Imaging 2010; 31: 192-203.

5. Cuadra MB, Cammoun L, Butz T, Cuisenaire O, Thiran JP. Comparison and validation of tissue modelization and statistical classification methods in T1- weighted MR brain images. IEEE Trans Med Imaging 2005; 24: 1548-1565.

6. Ashburner J. A fast diffeomorphic image registration algorithm. NeuroImage 2007; 38: 95-113.
